# Plant Allelochemicals as Sources of Insecticides

**DOI:** 10.3390/insects12030189

**Published:** 2021-02-24

**Authors:** Ivana Tlak Gajger, Showket Ahmad Dar

**Affiliations:** 1 Department for Biology and Pathology of Fish and Bees, Faculty of Veterinary Medicine, University of Zagreb, 10000 Zagreb, Croatia; 2 Division of Agricultural Entomology, KVK-Kargil II, Sher-e-Kashmir University of Agricultural Sciences and Technology of Kashmir, Srinagar 191111, India; showketdar43@gmail.com

**Keywords:** plant defense, allelochemicals, pesticides, insect herbivores, natural compounds

## Abstract

**Simple Summary:**

Due to the banning of many synthetic pesticides, current intensive farming systems require us to develop new approaches to integrated pest management. Devastating pests rarely occur in the wild and medicinal plants because of effective defense mechanisms. In contrast, only some of these defense mechanisms are found in cultivated crops. Biocidal compounds, derived from various wild and medicinal plants, are bioactive, biodegradable and constitute an ecological method for the successful management of insect pests. Therefore, an extensive study of various wild crops and some weeds is essential to identify new and potential plant species with insecticidal compounds.

**Abstract:**

In this review, we describe the role of plant-derived biochemicals that are toxic to insect pests. Biotic stress in plants caused by insect pests is one of the most significant problems, leading to yield losses. Synthetic pesticides still play a significant role in crop protection. However, the environmental side effects and health issues caused by the overuse or inappropriate application of synthetic pesticides forced authorities to ban some problematic ones. Consequently, there is a strong necessity for novel and alternative insect pest control methods. An interesting source of ecological pesticides are biocidal compounds, naturally occurring in plants as allelochemicals (secondary metabolites), helping plants to resist, tolerate or compensate the stress caused by insect pests. The abovementioned bioactive natural products are the first line of defense in plants against insect herbivores. The large group of secondary plant metabolites, including alkaloids, saponins, phenols and terpenes, are the most promising compounds in the management of insect pests. Secondary metabolites offer sustainable pest control, therefore we can conclude that certain plant species provide numerous promising possibilities for discovering novel and ecologically friendly methods for the control of numerous insect pests.

## 1. Introduction

Insect communities have positive and negative interactions with a range of plants in different climate zones [1,2]. Negative interaction with insects, which causes damage to the plants, has led to the development of various chemical complex defense mechanisms [3]. This plant diversity and evolution determines insect diversity. In particular, secondary plant metabolites, called allelochemicals, play a crucial role in plant–insect interactions [4].

According to the estimate, for the year 2050, a twofold increase in food production will be necessary to meet global demand [5]. Yield loss caused by arthropod pests is one of the reasons for less intensive production [6]. On a worldwide scale, the annual yield loss exceeds 15% [7,8]. The extensive use of pesticides causes a number of serious problems, including non-target effects on humans and beneficial organisms, including insect pollinators [9], natural enemies, pest resurgence, the emergence of secondary pests, biotypes, high costs associated with both active ingredients and the application and development of resistance to pesticides by target pests [10].

Plants respond to herbivory through intricate and dynamic defense systems. Induced defense response is one of the most important components of pest insect control. Damage caused by insect bites induce calcium ion fluxes and phosphorylation cascades, as well as systemic and jasmonate signaling [3]. As a result, plants produce a range of defensive metabolites to protect themselves against herbivores [11]. Defensive metabolites can be either stored as inactive forms, called phytoanticipins, or induced as phytoalexins for active defense response [3,4]. These bioactive compounds repel or intoxicate the insects and have a negative impact on their digestion. Carbon (C)- or nitrogen (N)-based anti-herbivore defense acts as a repellent, deterrent or growth inhibitor or causes direct mortality [4,12]. Plants have systemic resistance achieved through jasmonic acid, ethylene and salicylic acid (SA) pathways, leading to the biosynthesis of defensive proteins against herbivore pest insects. The stunning array of chemical- and protein-based defenses aims to detect invading organisms and inhibits them before they cause extensive damage [10].

This article provides an overview of common biochemical defense mechanisms of plants against insect pests. Additionally, some chemical characteristics responsible for insect pests resistance are described.

## 2. Mechanisms of Plant Defense against Insect Herbivores

Plants and insect pests are in constant interaction. Plants offer them food, a place for oviposition and shelter [13]. However, plants have also evolved various resistance mechanisms to overcome the damage done by insect pests [14,15]. According to the theory of host plant resistance of Painter [16], plant resistance against insects is defined as “the sum of the heritable qualities which influences the ultimate degree of damage done by the insect pest”. The plant resistance mechanisms that affect insects are constitutive or induced. They can be grouped into three main categories: antixenosis or non-preference, tolerance and antibiosis. The latter means that plants adversely affect the physiology of an insect, such as its survival, development and fecundity [17]. The adverse effect of antibiosis may be mild or cause death, including larval mortality, disturbance of the life cycle and the reduction in fecundity and fertility of the insect. Oyetunji [18] concluded that antibiosis is the main source of resistance in rice against the rice gall midge.

Broadly speaking, plant resistance against insects can be grouped into two categories. The first one is constitutive resistance, which includes the inherited ability of the host plant to defend itself against the insect pests, regardless of biotic or abiotic factors. The second is induced resistance, which appears as a response to attack by insect herbivores, diseases or abiotic factors [19]. Constitutive and induced resistance can be direct or indirect. In direct resistance, both morphological traits and secondary metabolites act as direct defense strategies to resist insect herbivores. In indirect resistance, plants rely on natural enemies of the herbivores to protect them. Herbivore-induced plant volatiles (HIPVs) emitted upon an insect damage are known to provoke indirect resistance. The HIPVs attract predators and parasitoids [20], which reduce the damaging caused by insect pests.

Anti-herbivory compounds are secondary metabolites of plants suppressing herbivore insects [21]. They can be divided into several subgroups: nitrogen compounds including alkaloids, cyanogenic glycosides and glucosinolates [22], terpenoids and phenolics [23,24]. The diversity of angiosperms during the Cretaceous period is associated with the sudden increase in speciation in insects [25]. Parallel to their evolution, selective biochemical processes in plants resulted in defensive adaptations against insect herbivores [6]. First, insects bit or chewed on plants. However, the coevolution of vascular plants and insect species caused new patterns of feeding to emerge, such as sap sucking, leaf mining, gall forming and nectar feeding [26,27].

Insect herbivore species greatly vary in their ability to cope with multi-faceted plant defense mechanisms. This speciation has driven the evolution of different host plants and food plants [27]. In the course of evolution, plants have developed many resistance mechanisms to reduce the damage caused by insects [28]. Insect adaptations to this defense are mostly related to their biochemical traits [29]. Plants’ defensive morphological characteristics, such as waxy cuticles, spines, setae, trichomes, thorns, toughened and hardened leaves (sclerophyll), granular minerals in plant tissues and divaricated branching, interfere with movement, feeding, oviposition and the reproduction of insects [30].

Plants have complex defense mechanisms against various insect feeding strategies [3,31]. A widespread opinion that specialist groups of herbivores are immune to the defense mechanisms of host plants is incorrect. Nevertheless, physiological adaptations of specialist insects cope with plant defenses. Specialists that rely on plant secondary metabolites as attractants and feeding stimulants can be negatively affected by plant defenses, in some cases simply via energy that is required for detoxification [32]. However, on average, specialist herbivores are less negatively affected by defense compounds than generalists. There is a long-standing paradigm that specialist and generalist herbivore insects interact with plants in well-defined ways [33]. For example, parsnip webworms (*Depressaria pastinacella*) eat furanocoumarins [34]; oleander aphids (*Aphis nerii*) consume jasmonic acid on sandhill milkweed (*Asclepias tuberosa*) [35]; monarch caterpillars (*Danaus plexippus*) eat jasmonic acid and SA containing sandhill milkweed (*Asclepia syriaca*) [35]; tobacco hornworms (*Manduca sexta*) eat nutriments containing nicotine [36]. However, cabbage caterpillars (*Pieris rapae*) are poisoned by isothiocyanates [37]. In members of the family Brassicae, glucosinolates were in higher concentration in flowers than leaves. Sinigrin was by far the most abundant glucosinolate compound both in leaves and flowers compared to 4-hydroxyglucobrassicin. Therefore, second- and third-instar *P. rapae* caterpillars prefer to feed on flowers. The higher concentrations of glucosinolate provide a nutritional benefit to the *P. rapae* in terms of higher growth rate [38]. In all of the above cases, specialists have a physiological adaptation to cope with the defense mechanisms of plants. It seems that just a small number of insects are immune to the deleterious effects caused by plant toxins. Specialist insect pest species sequester toxic chemicals and use them to protect themselves from predators.

In this context, Yactayo-Chang et al. (2020) suggested that digestibility reducers should be effective against all insects, although toxins can be overcome by specialists [39]. In some special cases, both generalists and specialists can overcome some digestibility reducers [39] to maximize their fitness [33]. In addition, some generalists possess remarkable abilities to consume highly toxic plants [40]. For example, cardenolides are bitter-tasting steroids present in the cells of milkweed, and they affect insects by disrupting the sodium and potassium flux. However, specialists such as *D. plexippus* have evolved physiological adaptations for tolerating these steroids [41,42]. Their larvae face an interesting trade-off: feed only on plants containing cardenolides, sequester cardenolides as anti-predator defense. However, high levels of cardenolides have negative effects and can kill early instar larvae [42]. These chemicals are constantly produced and stored in plants, following the damage by several species of chewing insects. That being said, even highly specialized insects are not entirely immune to the negative influence of secondary plant metabolites, such as cardenolides [43]. The generalists are typically more sensitive to plant toxins than specialists. Generalists suppress induced plant responses and specialists minimize the induction of high levels of protection. The induction of indirect defenses, such as extrafloral nectar and parasite-attracting volatile organic compounds (VOCs), is strong if the specialist is not actively sequestering toxins.

## 3. Plant Metabolites and Their Insecticidal Activity

Plant metabolites can be grouped into primary and secondary categories. Primary metabolites are substances directly involved in the growth, development and reproduction of all plants. These metabolites do not possess a defensive role. Secondary metabolites have a major role in defense against insects [23,44,45,46]. Compounds, such as phenol, tannin, peroxidase, polyphenol oxidase and Bt proteins (insecticides produced by bacterium *Bacillus thuringiensis*) can suppress insect populations [47,48]. 

According to D’Addabbo et al. [49], compounds such as alkaloids, phenolics, cyanogenic glucosides, polyacetylenes and polythienyls show biocidal activity. These compounds are often produced as by-products during the synthesis of primary metabolic products [50,51]. For example, geranium produces a unique chemical compound, called quisqualic, in its petals to defend itself against Japanese beetles (*Popillia japonica*) by paralyzing them within a period of 30 min [25].

Some of the metabolites, called phytoanticipins, are always synthesized in plants. They activate constitutive resistance against the corn earworm (*Helicoverpa zea*) [12]. Disparate metabolites are produced just after initial damage due to the induced ability to counteract *Helicoverpa armigera* and *Spodoptera litura* [48,52,53]. Additionally, it was found that infested cotton plants showed a higher level of defensive proteins (e.g., proteinase inhibitors, proline-rich proteins, lipoxygenase) than other plants after initial infestation with insect pests [54]. Induced defense is based on mobile metabolites with a relatively low molecular weight produced at low metabolic costs and only during or after insect attacks. However, compounds such as terpenoids, aromatics, and fatty acids have high molecular weight and are produced after insect invasion [46]. Quantitative metabolites are high in quantity, and their higher proportion in the diets of herbivores causes reduced feeding activity [55].

A more suitable and novel approach needs to be developed for insect pest management programs [56]. Plant allelochemicals based on plant–insect interactions are either innate or are C- or N-based. They can act as repellents, deterrents, growth inhibitors or can cause direct mortality [57,58]. As a result, insects have evolved strategies, such as avoidance, excretion, sequestration and degradation, to cope with these toxins (Table 1). This coevolution is based on the competition between insects and plants and finally leads to speciation [4]. Insect herbivores feeding on a plant species encounter potentially toxic substances with relatively non-specific effects on proteins (enzymes, receptors, ion-channels and structural proteins), nucleic acids, secondary metabolites, bio-membranes and specific or unspecific interactions with other cellular components [59,60].

Due to the antifeedant effect of biochemicals and their growth regulation effects [61], it is experimentally proven that neem-based insecticides show aversive effects on insect physiology [62]. In lepidopteran larvae, terpenes from neem have stimulatory effects on chemosensory receptor cells and affect the receptors in other organs [63]. Essential plant oils may be neurotoxic or may act as insect growth regulators and disrupt the normal process of morphogenesis [64]. For example, some monoterpenoids (D-limonene, myrcene, terpineol, linalool and pulegone), known as the main components of essential oils, have been used against various pests [65]. The toxicity of the ten most abundant monoterpenes of *Pinus contorta* against mountain pine beetles suggest that (−)-β-phellandrene, (+)-3-carene, myrcene, terpinolene, enantiomers of α-pinene, β-pinene and limonene caused mortality [66]. The monoterpene profile of plants showed a consistent foliar pattern over the growing season with δ-3-carene present in spring, whereas bornyl acetate increased during the growing season. In addition, these compounds were highly toxic for pulse beetles (Himachallol and β-Himachalene) [67]. Some plant oils are neurotoxic when insects feed on them. The most prominent symptoms are hyperactivity, hyper-excitation, followed by rapid knock down and immobilization [68].

Herbivorous insects use different physiological strategies to tolerate noxious and unpalatable toxins. These mechanisms include the involvement of carbohydrates that cover the unpalatable taste of toxins, extended dietary exposure to some unpalatable secondary plant compounds and dietary exposure to toxic compounds that induce the production of P450 detoxication enzymes. Therefore, herbivorous insects utilize an integrated suite of physiological mechanisms to detect potentially toxic compounds in foods and then selectively adapt to those that do not pose a serious threat to their growth and survival [69].

### 3.1. Alkaloids

There are numerous plant alkaloids, such as nicotine, caffeine, morphine, colchicine, ergolines, strychnine, scopolamine and quinine [70]. Alkaloids can affect nerve transmission in insects, disturbing the cell membrane and cytoskeletal structure, causing the collapse and leakage of cells [71]. For humans, the presence of alkaloids leads to bitter taste, whereas for specialist insects, it can be aversive or a feeding stimulant [72].

#### 3.1.1. Pyrrolizidine Alkaloids

Pyrrolizidine alkaloids (PAs) mediate plant defense in the form of feeding repellents. However, they are also toxic to intestinal microbes of general insect herbivores [73,74]. Jacobine and erucifoline are the most effective PAs against insect herbivores [75]. From a toxicity point of view, the structure of PAs is significant for their activity against insect pests. PAs, belonging to the senecionine type [76], contain the compound senecionine N-oxide, which elicits a toxic effect against *Spodoptera exigua*. Other PAs that occur in Senecio are seneciphylline, jacobine and senkirkine [77]. Each species of this genus usually contains multiple PAs and has a species-specific PA structure [76]. PAs are not induced in shoots after herbivore attack but during the damaging of the roots [78]. PAs can occur in two configurations, the tertiary free base and the N-oxide. In the roots, the PAs are almost exclusively present as N-oxides. Approximately 35% of the PAs are tertiary free bases. In *S. exigua* mid-gut, N-oxide can be broken to the tertiary PA, which is absorbed and further reduced to highly unstable toxic pyrroles. The N-oxide of PA is non-toxic, unable to passively pass through the membranes and cannot be directly converted into toxic pyrroles [79]. PAs are composed of different bases. PAs are likely to be broken down to toxic pyrroles by P450 enzymes in the insect’s gut [80]. The generalist caterpillar *Spodoptera littoralis* excrete PAs very effectively, thus successfully avoiding intoxication. Senecionine N-oxide is passively absorbed in the hemolymph and easily reduced to the tertiary alkaloids in the gut of *S. littoralis* [76,81].

#### 3.1.2. Nitrogen Compounds

**Figure 1:**
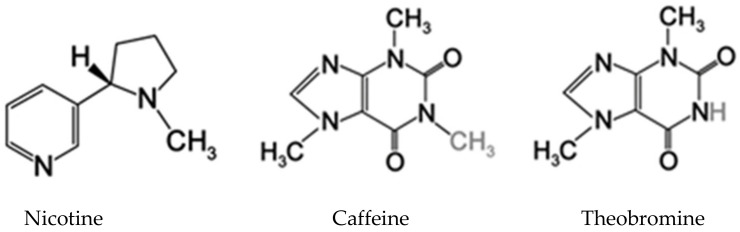
Bitter tasting nitrogenous compounds: nicotine, caffeine, theobromine.

Bitter tasting nitrogenous compounds can be found in many vascular plants, including caffeine, cocaine, morphine and nicotine [82], derivates of aspartate, lysine, tyrosine and tryptophan. Many of these substances are known to elicit aversive or toxic effects in insects (Figure 1).

The toxicity of nicotine is one of the important defense mechanisms against a range of insects. The direct contact of insects with nicotine leads to paralysis and, eventually, death [83]. Tobacco keeps spiders away, but the tobacco caterpillar has managed to overcome this plant defense. However, when tobacco leaf is wounded, plants immediately release a “bouquet” of distress chemicals, known as green leaf volatiles (GLVs), containing long fatty acid chains as a sort of defense against the damage caused by tobacco caterpillars [84].

Members of the nightshade family, e.g., brinjal (*Solanaceae* spp.) produce atropine, an alkaloid that is neurotoxic and known as a cardiac stimulant [85]. Nicotine is produced and stored in vacuoles. It is released when insects feed on the leaves and break vacuoles.

### 3.2. Terpenoids

The largest group of secondary metabolites that are involved in the defense mechanisms of plants include naturally occurring hydrocarbons, terpenes [86,87]. This group is found in all plants and represents a huge class of more than 22,000 compounds (e.g., Table 2 and Table 3). In this group, isoprene units form the backbone of terpenes. Terpenes are mainly biosynthesized in the 2-c-methyl-1-D-erythritol-4-phosphate (MEP) pathway. Besides insect toxicity, terpenes also contribute to the fragrance of plants [88]. The volatile gases emitted during photosynthesis are actually the simplest terpenoids.

In most of the coniferous trees, defense against insect pests relies on terpenoids and polyphenols that are accumulated in the resin canals of the xylem [89]. However, these defense compounds are equally distributed throughout the roots and shoots [90]. Terpenoids consist of different numbers of isoprene units, e.g., monoterpenoids (two units), sesquiterpenoids (three units), diterpenoids (four units) and triterpenoids (six units). Plant steroids and sterols are produced on the basis of vitamin D or glycosides precursors [71]. However, many other factors facilitate the synthesis of terpenes in the course of evolution. For example, biotic partners, pollinator mutualism, geographic distribution and terpenoid latex production.

Additionally, terpenes defend plants against insect herbivores indirectly by enhancing the effectiveness of natural enemies of the herbivores. This is achieved with the releasing blend of specific volatiles. Such communication with the environment attracts beneficiaries (e.g., insect pollinators and seed dispersers), including predators, parasitoids, and herbivores [91].

#### 3.2.1. Monoterpenoids and Sesquiterpenoids

Monoterpenoids and sesquiterpenoids are highly volatile and protect plants from attacks of pest species. Individual and/or combined resin volatiles are present in hemlock (*Tsuga canadensis* L.), where it mediates resistance to woolly adelgid infestations [92]. This infestation also results in benzyl alcohol and methyl salicylate accumulation. When conifers are attacked by insects or pathogens, they increase the content of monoterpenes and sesquiterpenes [93]. Similarly, a large amount of the monoterpenoid menthol and menthone is produced by mint plants (*Mentha* spp.). These compounds are stored in glandular trichomes of the epidermis of the mint plant. Monoterpenoid esters, known as pyrethrins, are produced by chrysanthemum plants and are neurotoxic for insects. Many commercially available neurotoxic insecticides are synthetic counterparts of pyrethrins, also called pyrethroids (permethrin and cypermethrin). Many spices, seasonings, condiments and perfumes are made using monoterpenoids. Monoterpenoids are toxic for insects but relatively harmless to humans. For instance, peppermint and spearmint (*Mentha* spp.), basil (*Ocimum* spp.), oregano (*Origanum* spp.), rosemary (*Rosmarinus* spp.), sage (*Salvia* spp.) and savory (*Satureja* spp.) are toxic for insects [93]. Terpenes obtained from orange oil (*Chenopodium ambrosioides*) and neem oil are used as biopesticides with promising results for the control of aphids in green houses.

#### 3.2.2. Diterpenoids

Diterpenes and carotenoids are produced in the non-mevalonic acid pathway in plastid organelles [94]. In cotton (*Gossypium hirsutum*), gossypol contains isoprene units that can be found in latex and resins. These units are quite toxic to insects and act as a feeding deterrent [95]. Diterpenes are responsible for poisonous leaves of the Rhododendron. The two metabolites, rhododendron and romedotoxin, are present in all plant parts. Romedotoxins secreted in nectar are Na+ channel inhibitors for thrips insects [96]. Spinach (*Spinacia oleracea*) disrupts larval enlargement and maturing and also causes insect death by producing the phytoectysones. Furthermore, the fresh scent of lemon and orange peels belongs to a class of triterpenoids, called limonoids, which act as insect deterrents. However, the limonene terpene is a key compound in citrus fruits, responsible for insect attraction, and plays an important role in the context of pollination. Azadirachtin and Citronella are very powerful limonoids that have been isolated from neem trees (*Azadirachta indica*) and lemon grass (*Cymbopogon citratus*). Citronella contains high levels of limonoids and has become a popular insect repellent. Moreover, citronella is biodegradable and has low toxicity for humans. Metabolites from *A. indica* exert a strong insect repelling effect and also act as a feeding deterrent. It contains a-pinene (3%), camphene (2.12%), chrysanthenyl acetate (10.6%), borneol (8.07%), camphor (6.54%), a-phellandrene (1.05%) and p-cymene (1.15%) [97].

#### 3.2.3. Saponins

Saponins are glycosylated triterpenoids present in the cell membranes of numerous plants. This group acts as a detergent that leads to the disruption of the cell membrane, causes cell death, and ultimately kills insect pests [98]. The insecticidal activity of saponins is mediated via an interaction with cholesterol, which disrupts the synthesis of steroids from ecdys [99]. Most legumes contain saponins and show insecticidal effects (repellent/deterrent). The most often observed effects are increased mortality, lower food intake, weight reduction, retardation in development and decreased reproduction [100]. Consequently, these useful plant components pave the way towards a new strategy for protecting crops in modern agriculture and horticulture against insects, either by spraying or by selecting high saponin-containing varieties of commercial crops. In deserts, the shrubs of the genus Acaciacontain contain high concentrations of saponins in seed pods in order to prevent birds from eating the seeds. Fruits of *Sapindus mukorossi*, leaves of *Cestrum nocturnm*, *C. diurnum* and *Asclepias curassavica* are rich sources of saponins that can be used as mosquito larvicide [101].

### 3.3. Phenolics

**Figure 2:**
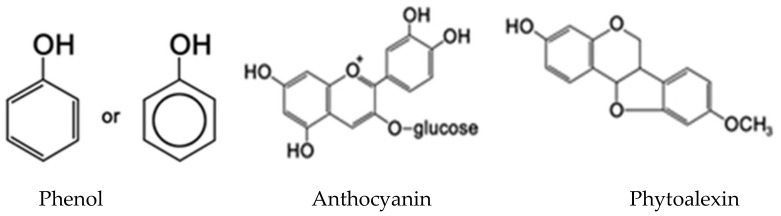
Plant phenol compounds and their derivatives: phenol, anthocyanin, phytoalexin.

Phenolics are secondary metabolites that include several classes. They are structurally diverse, arising from the shikimate-phenylpropanoids-flavonoids pathways, and consist of an aromatic six-carbon ring bonded to a hydroxyl group. Plants require phenolic or phenol compounds (Figure 2) particularly for resistance to insects [43,48,106,107] and pathogens. Plants, unlike animals, cannot rely on physical mobility to escape their predators, thus, synthesis of many phenolic compounds is a useful defense mechanism against the crop pests [108]. Some phenols exert antiseptic properties, while others disrupt the insect’s endocrine activity. Prophenoloxidase (PPO) is an important innate immune protein in plants, which is involved in the cellular defense [54,109].

Phenols can be in a form of the simple tannins or more complex flavonoids. Lignin, silymarin and cannabinoids are the main samples of phenolics used for defense in plants. Phenolic compounds are classified as shortly and widely distributed, and as polymers. Due to their location in plant and their chemical structure, the insoluble phenolic compounds are not digested in insect mid-gut and may be partially or fully recovered in the feces. Simultaneously, soluble parts can cross the intestinal barrier and can be found in the blood, native or as metabolites [110]. Phenolic compounds have antifeedant, toxic and regulatory activity affecting insect physiological processes or repel the phytophagous insects [111]. They promote oxidative stress in aphids and tissues [112] and were reported as a resistance factor in mango and brinjal against *Bactrocera dorsalis* [113], *Leucinodes orbonalis* [43] and *Spodoptera litura* [54].

### 3.4. Tannins

Tannins are condensed polymers consisting of polyhydroxyphenols and polyflavonoids made up of two to 50 molecules. They are prone to oxidization in insects under high alkaline conditions, forming semi-quinone radicals and quinones, which, at higher concentrations, cause toxicity. The tannin classes including ellagitannins and gallotannins commonly occur in many plant species and cause insect toxicity.

Silica and lignin are constituent elements of the cell walls of plants. They are rigid, insoluble and indigestible for insects. Due to these characteristics, they can grind down the mandible of insects [43,114]. In this context, betulinic acid was found to have very high antifeedant activity against all stages of the *Papilio demoleus* [115].

Many compounds containing flavonoids, anthocyanins, phytoalexins and furanocoumarins are phenol derivatives. Furanocoumarins are highly toxic to insects and many other organisms due to their integration into DNA, leading to rapid cell death [114].

Anthocyanins and phytoalexins act as insect growth inhibitors, which are mediated by the limited assimilation of dietary proteins, the inhibition of digestive enzymes and delayed development [43,116]. Anti-herbivore defenses based on phenol derivatives against insects act as repellents, deterrents and growth inhibitors. Moreover, they can cause immediate death when exceeding a critical dose. In response to these compounds, insects have evolved strategies such as avoidance, excretion, sequestration and the degradation of tannins. These interactions have given rise to the co-evolution and co-diversification between insects and plants [4].

### 3.5. Salicylic Acid

SA consists of an aromatic ring bearing a hydroxyl group. This acid is defensive in action [117]. The mid-gut digestive and detoxifying enzymes of insect pests are defensive against adverse effects mediated by SA. Thus, SA is involved in various metabolic plant processes, such as lignin biosynthesis, the regulation of responses to abiotic stress, allelopathy and pest resistance [118]. In some plants, such as *Arabidopsis thaliana,* SA perception plays a significant role in disease resistance, through activity of its five paralogs of master regulator NPR1 (nonexpresser of pathogenesis related 1) [119]. It is reported that SA induces resistance in groundnut against *H. armigera* [53] by affecting larval survival and the activity of digestive serine protease and trypsin enzymes. During sap sucking by aphids, plants release growth regulators, jasmonic acid and SA, which act as defense molecules [120]. Furthermore, in plants, SA and jasmonic acid signaling pathways are commonly known to mediate induced defense responses by expressing the negative crosstalk between host plants and herbivore insects [121].

### 3.6. Lignin

Lignin is the second most abundant polymer, after cellulose, found in nature. Among the many roles that lignin plays in plant growth and development, the most important are structural support and resistance to biotic [122] and abiotic stress. Lignin is the end product of the phenylpropanoid pathway and a heteropolymer of three-hydroxycinnamyl alcohol monomers or monolignols: p-coumaryl alcohol, coniferyl alcohol and sinapyl alcohol [123]. Hydroxycinnamates act as precursors of different polyphenolic compounds [11]. Monolignols and their precursors are synthesized in the endoplasmic reticulum and later transported as monolignol-glucosides to the cell wall, where lignin is deposited. Lignin is a highly branched heterogeneous polymer found in secondary cell walls [72], consisting of hundreds or thousands of phenolic monomers, and it is insoluble, rigid and almost indigestible. It provides an excellent physical barrier against insect attacks [124].

Plant tissue toughness is one of the key factors that reduces the damage in plants [22,125]. The tougher the tissue, the higher the lignin content. Tissues that contain high concentrations of lignin are unpalatable for insects. Increased lignin deposition might have additional negative effects on insects because phenoloxidase enzymes are involved in the polymerization of lignin, and this generates toxic by-products such as quinones and peroxides [126,127]. In maize, compounds namely, maysin, chlorogenic acid and phenolic acids [128] are biosynthetically related to insect defense. When the integrity of plants is injured by insects, or when plants suffer from pathogen infections, they start with the lignification of their cell walls. In such a case, lignin seems to act as a chemical or physical barrier to protect the remaining plant tissue from further damage [129,130]. During biotic stress, plant cell walls also exploit sophisticated sensing mechanisms to maintain wall integrity [131].

### 3.7. Glycosides, Defense Proteins and Enzymes

Cyanogenic glycosides are produced by plants and great efforts are currently undertaken to enable their artificial synthesis for insect pest control, whereas in other crops, efforts are made to improve food safety by their removal [132]. Enzymes, such as glycosidases and hydroxyl nitrile lyases, convert cyanogenic glycosides into hydrogen cyanide (α-hydroxynitriles). Hydrogen cyanide is stored in various compartments of tissues within the plant and cause toxicity to generalist insect herbivores [132,133]. Glucosinolates are sulfur-containing compounds that serve as repellents for various insect pests. Glucosinolates are converted into isothiocyanates during enzymatic metabolism. This group has pungent and irritating smell, thereby stopping insect feeding activity [134].

Furthermore, the oxidation state of secondary plant metabolites is associated with resistance to insects [135]. Reactive oxygen species are an important component of the immediate response of plants to insect damages [136,137]. Proteinase inhibitors reduce the proteolytic activity of insects’ mid-gut enzymes and decrease the availability of amino acids for absorption. Peroxidase and protein content in leaves and seeds of faba bean (*Vicia faba*) significantly affect insect infestations [138]. Polyphenol oxidase also regulates insect feeding activity, growth and development and plays a leading role in plant defense [135]. Plants have proteinase inhibitors, which delay larval development without directly causing mortality [139]. Proteinase inhibitors from *Madhuca indica* seeds negatively affect the development of *H. armigera* [140]. In contrast, a Kunitz-type serine protein inhibitor from the *Butea monosperma* acts as a competitive inhibitor. It retards growth and development and affects the fecundity and fertility of *H. armigera* [141].

Protein inhibitors are often enriched at sites where an insect attack is most likely to happen—for instance, in plants seeds, bulbs and leaves. In sugarcane, trypsin inhibitors are present in leaves, lateral buds and seed tissue. The bi-functional α-amylase-trypsin inhibitor is found in plant tissues, such as stem and its bark, apical meristem, as well as in leaves. It inhibits midgut α-amylase activity with negative effects on the growth rate of *H. armigera*, suggesting its suitability for insect pest control [142].

The immediate response of plants to the contact of insects leads to unbalanced ion flux across the cell membrane at the damaged site. Thus, a difference in charge leads to a transmembrane potential change that induces signal transduction and the generation of reactive oxygen, nitrogen species and other defensive inhibitors [143]. In other words, insect damage of the plant results in an increase in chemical inhibitors [144]. Further, calcium signaling, cation channel activity and the formation of secondary messengers released by the damaged tissue may also help the host plant to defend itself against insect pests [145]. This was also demonstrated in a transgenic tobacco, where a cowpea trypsin inhibitor gene has enhanced levels of resistance to a variety of insect pests [146,147]. Similarly, cysteine proteinase inhibitors were detected in various fruits and cereals with the highest expression in storage organs such as seeds, stems and leaf–root transition zones. In China, cysteine protease inhibitors were used for *Chilo suppressalis* management by targeting the digestive cysteine proteases or through RNA interference-based silencing of cysteine proteases, which disrupts the developmental regulation of insect pests [2].

Seeds contain special proteins that inhibit insect pest proteolytic enzymes by forming complexes, blocking active receptors and by changing enzyme structures. All this leads to the decreased or complete interruption of proteins digestion in insects. For example, in piegonpea and pea nuts, the seeds storage of proteins and inhibitors (α-amylase/trypsin inhibitor) suppress the activity of the gut enzymes of *H. armigera* to protect the seed tissues from damage [148]. These seed proteins are generally small and contain the amino acid cysteine [149]. Research suggested that α-amylase inhibitors contained in *Amaranthus retrofluxes* seed extracts have good defensive potential and, thus, can be used in the management program for suppression of the *Ephestia kuehniell* [150].

Defensins are found in all types of plant tissues including leaves, pods, tubers, fruits, roots, the bark and floral tissues, where it causes a range of biological activities. Some defensins inhibit digestive proteins in insects. Digestive enzyme inhibitors are proteins that block the normal digestion and absorption of nutrients by insects. Alpha-amylase inhibitors are proteins commonly found in legumes that inhibit starch digestion. Insects feeding on legumes activate a chain of molecular signaling events inducing a systemic production of these compounds in distal plant tissues. This contributes to the protection of undamaged plant parts against subsequent insect bites. The substrate specificity and the exact mechanism of the plant protein still need to be clarified, as well as the characterization of the three-dimensional structure of this protein [151].

## 4. Specialized Defense Mechanisms

Idioblasts, also called “crazy cells”, help to protect the plants against the insect pests. They contain oil, latex, gum, resin, tannin and pigments. Some of them contain mineral crystals and poisonous calcium oxalate or carbonate or silica. When the terminal end of the cell is broken by water pressure, calcium oxalates are released, resulting in the stiffening of the tissue structure [152]. Furthermore, sharp oxalate crystals tear the mouth parts of the insects during the feeding activity. Pigmented cells often contain bitter-tasting tannins, making plant parts undesirable as food sources [153], but interestingly, some insects utilize them for growth and development. Sclereids denote irregular-shaped cells with thick secondary walls that are difficult to chew [85]. For example, the rough texture of pear fruit (*Pyrus* spp.) contains thousands of sclereid stone cells, which can abrasively damage the teeth of animals. Stinging nettles (*Urtica dioica*) produce stinging cells characterized by sharp point like hypodermic needle that breaks off during feeding and injects “acrid fluid”, containing highly irritating toxins. Crystalliferous cells damage insect mouth parts after chewing the abovementioned plants, and they can be toxic for insects after digestion.

## 5. Extraction, Application, Registration and Market Availability of Plant-Based Products

The extraction of plant secondary metabolites includes solvent extraction processes [154]. Successful extraction starts with the selection and preparation of plant samples that are significant for the extraction of bioactive compounds. In this process, it is important to reduce the interference with other compounds that may coexist with targeted compounds. The contamination of the extract may lead to the decomposition and dissociation of valuable metabolites. Besides classical extraction techniques, different sophisticated methods and procedures have been designed [155]. The phytochemical extraction processes of various metabolites include “acid-base shakeout” (alkaloids), maceration (tannins), ultrasound assisted extraction (phenols), followed by gas chromatography (terpenoids), high-performance liquid chromatography (SA), Fourier transformed infrared spectroscopy, thermo-gravimetric analysis, differential scanning calorimetry (lignin), microwave assisted extraction (anthocynin), cold press, and soxhlet methods (azadirectin) [155].

Today, there is an increased demand for effective organic pesticides with high selectivity [156]. The botanicals are completely biodegradable into nontoxic final products suitable for their use in crops pest control (Table 4).

Stakeholders, policy makers, distributors and end users are involved in the development and commercialization of the plant-based pest control products [60]. Despite the intense worldwide collaborations for the promotion of biopesticides, their use in agriculture is still limited, and many issues linked with their use need to be resolved. The market is often in disagreement with the legislation framework, as well as with general and scientific opinions. Consequently, farmers are often confused, since the plant-based products have short persistence in the field. However, the latest techniques, such as nano-formulations and microencapsulation can improve the stability and residual activity. Additionally, the better regulation, commercialization and availability of low-risk compounds enhance their market and utilization. Legislation and industry should give production support to small- and medium-sized companies to easily meet the expectations of end users, as well as ensure a market for products [162].

For many years, plant-based pesticides have been substantially evaluated and registered on the model for the registration of standard pesticides. Today, registration protocols for biopesticides are being reformed and modified by different respective organizations, at the international, national and regional levels. In the USA, the “Environmental Protection Agency (EPA)” supervises the regulation of biopesticides. In the European Union (EU), botanicals are widely available in the form of food supplements. However, the registration procedure for biopesticides is much longer and more complex compared to other developed nations. In the EU, registration of biopesticides is monitored by the “EU Plant Protection Regulation” (Reg. 1107/2009) in conjunction with the Regulation on Maximum Residue Levels (MRLs) in food—396/2005—and the Directive on sustainable use of pesticides; 2009/128/EC. Before the product is introduced to the market, the active ingredients are being approved on quality and safety for food, environment and human health (“risk-based approach”) [163].

Currently, biopesticides present only a small share (<5%) of the total crop protection products market. Globally, their market is expected to reach USD 5833.4 million by 2022, with the majority of registrations expected from the USA. More than 200 products are available in the USA market, compared to 60 analogous products in the EU (Table 5). In the USA, Canada and Mexico, more than 45% of the biopesticides are sold compared to Asia, which contributed only 5% to the world market [164]. Internationally, biopesticide use is increasing by almost 10% each year. In regard to the food safety and humans involved in pest treatment, it is of paramount importance that the global market of biopesticides further expands in the future, because eco-friendly products are effective alternatives to synthetic pesticides. With the annual growth rate of 15%, it is expected that biopesticides will share the same market with synthetics between the 2040s and the 2050s [165].

Over time, the acceptance of biopesticides by farmers has gradually increased due to the negative side effects of synthetic pesticides. Since the biopesticides are less effective and biodegradable, they have to be applied more than once for effective treatment. However, the frequent application of biopesticides, due to high costs, represents obstacles for farmers.

## 6. Side Effects on Non-Target Insects

The side effects of plant metabolites on beneficial insects, especially honeybees (*Apis mellifera*) as the main pollinator of cultivated plants, have remained unidentified for many years [166]. Compounds such as andiroba oil, garlic extract and neem oil applied at high concentrations show an acute toxicity to honeybee larvae. Larvae fed with the syrup containing these oils showed lower body mass in emerged young workers. Additionally, it is observed that garlic extract, neem oil and rotenone decrease the rate of locomotion activities in adult workers [167]. Therefore, new studies on the side effects of secondary plant metabolites on honeybees and other beneficial insects, should be conducted.

## 7. Conclusions and Future Perspectives

Current intensive farming demands new, effective and ecofriendly insect pest management. The use of plant secondary metabolites with insecticidal effects is one of the cornerstones of environmentally acceptable pest management strategies. Devastating pests rarely occur in nature, because wild plant populations contain a range of these metabolites, which provide an effective defense against the harmful insects. Unfortunately, many of these valuable plants and their metabolites have not yet been explored. Therefore, it is essential to conduct new studies on various wild plants with respect to their repellent and deterrent properties.

Furthermore, pest management, based on allelochemicals, is compatible with existing farming conditions. Consequently, the identification, extraction, bioassay, isolation, evaluation and persistence of botanicals need to be taken into consideration in future studies.

An increasing number of farmers sell organic products, despite the high costs associated with organic insect pest control. However, in many less developed countries, high costs of these products are not acceptable without governmental support. Therefore, new social studies should focus on local initiatives that enable farmers in developing countries to afford biopesticides.

Additionally, new research should investigate side effects of plant-based products on beneficial insects, especially bees.

## Figures and Tables

**Table 1:** Main groups of allelochemicals and their corresponding physiological effects on insects [50].

Allelochemicals	Behavioral or Physiological Effects
Allomones	Provide adaptive advantages to the producing organisms
Repellents	Orient insects away from the plant
Locomotor excitants	Speed up movement
Suppressants	Inhibit biting or piercing
Deterrents	Prevent feeding or oviposition
Arrestants	Immobilize insects
Digestibility reducing	Interfere with processes of food utilization
Toxins	Produce chronic or acute physiologic disorders

**Table 2:** Some terpenoids in plants and their activities in insect–plant interactions [63].

Chemical	Source Plant	Affected Pest Insect	Effect
α-Pinene			
3-Carene	*Pinus silvestris*	*Blastophagus piniperda*	Repellent
α-Pinene			
3-Carene	*Pinus taeda*	*Dendroctonus frontalis*	Attractant
α-Pinene			
3-Carene	*Pinus ponderosa*	*Dendroctonus brevicomis*	Attractant
		*Heliothis* spp.	
Gossypol	*Gossypium hirsutum*	*Epicauta* spp.	Feeding deterrent
		*Anthonomus grandis*	Feeding stimulant
		*Tetranychus urticae*	Feeding deterrent
Cucurbitacin	Cucurbitaceae	*Acalymma* spp.	
		*Diabrotica* spp.	Feeding excitant

**Table 3:** Different plant species with common volatile terpenes released in response to insects’ attack.

Plant	(E)-b Ocimene	Linalool	(E)-4,8-Dimethyl- 1,3,7-Nonatriene	(E,E)-a- Farnesene	(E)-b- Farnesene	(E,E)-4,8,12- Trimethyl 1, 3, 7, 11- Tridecatetraene	Ref.
Cucumber	+	−	+	+	−	+	[102]
Apple	+	−	+	+	−	+	[71]
Lima bean	+	+	+	−	+	+	[102]
Cotton	+	+	+	+	+	+	[103]
Corn	+	+	+	+	+	+	[104]
Tobacco	+	+	−	+	+	−	[105]
Potato	+	+	−	+	+	−	[81]

**Table 4:** Some secondary metabolites of plants and their effects on target insect pests.

Plant Species	Biochemical Released	Insect Affected	References
Wild pistachio trees (*Pistacia vera*)	(E)-β-ocimene (boci); (Z)-ocimene	Aphid *(Slavum wertheimae)*	[153]
Alder tree *(Alnus glutinosa)*	Lipoxygenase	Geometrid moth (*Cabera pusaria)*	[88]
Common bean *(Phaseolus vulgaris)*	(*Z*)-3-hexenyl acetate	Pea leaf miner *(Liriomyza huidobrensis)*	[88]
Arabidopsis (*Arabidopsis thaliana*)	Jasmonate	Cicada (*Cicadoidea* spp.)	[157,158]
Various plant species	Isoprene or/and monoterpene	Various herbivores	[159]
Various plant species	Aldehydes, alcohols	Various herbivores	[160]
*Brassicaceae* spp.	Glucosinolates	Diamondback moth *(Plutella xylostella)*	[161]
Brinjal (*Solanum melongena*)	Phenol, solcidene	Eggplant fruit moth *(Leucinodes orbonalis)*	[22]
Brinjal (*Solanum melongena*)	Anthocynin (chlorophyll)	Eggplant fruit moth (*Leucinodes orbonalis*)	[23]
Neem (*Azadirecta indica*)	Azadirectin	Various insects	[43]

**Table 5:** Commercial plant-based products available in the EU and USA markets, their active substances, origin and target pests.

Active Substance	Plant Source	Target Pest	Commercial Product
Pyrethrins	*Chrysanthemum cinerariifolium*	Effective against many insect orders	Azera Gardening BotaniGard^®^MAXX PyGanic Gardening Monterey Bug Buster-O Bug spray Persian Powder Pyganic Tersus
Rotenone	*Derris elliptica*	*Leptinotarsa decemlineata* *Diabrotica undecimpunctata* *Alticini* spp. *Hellula undalis* *Byturus tomentosus* *Crioceris asparagi*	Derris Cube Tuba
Sabadilla	*Sabadilla officinarum*	Effective against many insect orders	Red Dog Natural Guard Sabadilla 30c
Ryania	Salicaceae family plants	Effective against many insect orders	Ryanodine Natur Gro R-50 Natur Gro Triple Plus Ryanicide Ryan 50
Nicotine	*Nicotiana tabacum*	Effective against many insect orders, especially beetles	Golden leaf tobacco spray
d-Limonene	Citrus fruits	Effective against many insect orders	Limonene Bardac 22
Linalool	Flowers of different plant families	Effective against many insect orders	Bugx-30 Deet Ben S Natrapel Sting Relief
Neem	*Azadirachta indica*	Effective against many insect orders	Neem oil 70% Nimbio-Sys Aza-Direct AzaGuard Azatin Azatrol Azera Aztec TriAct Molt-X Neemix Ornazin
Capsicum oleoresin extract + Garlic oil	*Capsicum* spp. *Allium sativum*	Effective against many insect Orders	Captiva
Rosemary oil + peppermint oil + geraniol	Several plants of different plant families	Homoptera, Hemiptera, Lepidoptera, Arachnids: aphids, beetles, plant bugs, whiteflies, mites, thrips and caterpillars	Ecotrol Plus
Soybean oil	*Glycine max*	Earworm root worms and fall armyworm	Golden Pest Spray Oil
*Chenopodium ambrosioides* extract	*Chenopodium ambrosioides*	Diptera, Homoptera, Hemiptera: leafminers, thrips and whiteflies	Requiem
Trans anethole + thymol Thymol + citronellal Terpineol + citronellal	Various leaf and flower extract oils	*Spodoptera litura*	Binary mixture oil
Leaf extract	*Melia toosendan*	*Peridroma saucia*	Mixture oils
Linalool + 1,8-cineole linalool + terpineol or thymol 1,8-cineole + terpineol or thymol	*Cedrus* spp. *Lavandula angustifolia* *Mentha pulegium* *Eucalyptus* spp. *Citronella* spp.	*Chilo partellus*	Binary mixture oil
Peniocerol + macdougallin	*Myrtillocactus geometrizans*	*Tenebrio molitor*	Binary mixture oil
Methanol extract	*Fagopyrum esculentum*	*Myzus persicae*	Plant extract
Coumarins, monoterpenes + sesquiterpenes	Plant of family Apiaceae *Pinus sylvestris*	*Acanthoscelides obtectus* *Tribolium castaneum* *Lipaphis pseudobrassicae* *Hylotrupes bajulus*	Oils
Essential *oils* + *extracts*	*Ageratum conyzoides*	Effective against many insect orders, especially *Plutella xylostella*	Ageratum extract
Leaf extract	*Origanum vulgare*	*P. xylostella* *Trichoplusia ni*	Leaf extract

Data about commercial products names, active substances and their origin as well as use were taken from commercial websites and/or product labels.

## Data Availability

Data available in a publicly accessible repository.
